# Associations of Phase Angle, GLIM-Defined Malnutrition, and Rectus Femoris Ultrasound with 30-Day Mortality in Critically Ill Adults: A Prospective Cohort Study

**DOI:** 10.3390/nu18183059

**Published:** 2026-09-19

**Authors:** Alejandro García-García, Alejandro Úbeda-Iglesias, Helena Pozo-Romero, Nuria Trujillo-Garrido, María José M. Alférez, Eduardo Sánchez-Sánchez

**Affiliations:** 1 Biomedical Research and Innovation Institute of Cádiz (INiBICA), Puerta del Mar University Hospital, 11009 Cádiz, Spain; 2 Intensive Care Unit, Punta de Europa University Hospital, 11207 Algeciras, Spain; 3 Department of Nursing and Physiotherapy, University of Cádiz, 11009 Cádiz, Spain; 4 Department of Physiology, Institute of Nutrition and Food Technology José Mataix, University of Granada, 18071 Granada, Spain; malferez@ugr.es

**Keywords:** critical illness, phase angle, malnutrition, body composition, muscle ultrasound, mortality

## Abstract

**Background and aims:** Early phenotyping in the ICU is challenging because severity scores do not capture malnutrition, cellular integrity, or muscle reserve. We assessed whether phase angle (PhA), GLIM-defined malnutrition, and rectus femoris ultrasound were associated with 30-day mortality and whether continuous PhA added prognostic information beyond illness severity. **Methods:** We analyzed a prospective single-center cohort of adults admitted to a 12-bed ICU. Within 24–48 h, patients underwent bioelectrical impedance analysis, rectus femoris ultrasound, GLIM, mNUTRIC, CONUT, APACHE II, and SOFA assessment. Primary multivariable analyses treated PhA and rectus femoris cross-sectional area (CSA) as continuous variables; ROC-derived cut-offs were considered exploratory. Potential non-linearity was examined with restricted cubic splines, and the continuous PhA model underwent bootstrap internal validation. **Results:** Among 125 patients (63.2% men; median age 68 [60–76] years), 30-day mortality was 24.0% (30/125). GLIM-defined malnutrition was present in 78.4%, including 12.0% with severe malnutrition. Non-survivors had lower PhA (4.3 [3.6–4.8] vs. 4.8 [4.2–5.5]°; *p* = 0.008) and smaller rectus femoris CSA (2.6 [1.9–3.4] vs. 3.6 [2.5–5.0] cm^2^; *p* = 0.007). In the adjusted continuous PhA model, APACHE II (OR 1.23 per point, 95% CI 1.13–1.34; *p* < 0.001) and severe GLIM-defined malnutrition (OR 5.95, 95% CI 1.45–24.32; *p* = 0.013) remained independently associated with mortality, whereas lower PhA did not (OR 1.11 per 1° lower, 95% CI 0.74–1.69; *p* = 0.611). Adding continuous PhA to the reference model changed AUC from 0.868 to 0.867 and did not improve fit (likelihood ratio *p* = 0.607). Continuous CSA was also not independently associated with mortality (OR 1.17 per 1 cm^2^ lower, 95% CI 0.81–1.69; *p* = 0.412). **Conclusions:** APACHE II and protocol-defined severe GLIM malnutrition, but not continuous admission PhA or rectus femoris CSA, retained independent associations with 30-day mortality; the GLIM association should be interpreted in light of the protocol-specific morphofunctional operationalization of the muscle criterion. Lower PhA and CSA characterized non-survivors in unadjusted analyses; cohort-derived thresholds should be considered hypothesis-generating and require external validation.

## 1. Introduction

Nutritional risk emerges early during critical illness. Systemic inflammation, increased proteolysis, immobilization, and inadequate nutrient provision contribute to rapid skeletal muscle and nutritional deterioration during critical illness [1]. Current critical care nutrition guidelines therefore recommend early and repeated nutritional assessment together with individualized nutritional strategies [2,3]. However, pragmatic bedside tools able to accurately identify high-risk patients remain limited [4,5].

A major limitation in routine ICU practice is that conventional severity scores estimate acute illness severity but do not directly assess current malnutrition or nutrition-related risk. The Global Leadership Initiative on Malnutrition (GLIM) criteria provide a structured framework to identify established malnutrition [6], whereas the modified Nutrition Risk in the Critically Ill (mNUTRIC) score identifies patients at higher nutritional risk during critical illness [7,8]. In ICU cohorts, GLIM has shown feasibility in critically ill populations [9], and combining GLIM with nutrition-risk screening tools may improve prognostic stratification [10,11].

Bedside body composition tools may offer clinically relevant information not captured by conventional severity scores. Bioelectrical impedance analysis (BIA) is a rapid, non-invasive bedside technique that provides measures related to hydration status, lean mass, and phase angle (PhA) [12]. PhA, derived from resistance and reactance, is regarded as an integrated marker of cell membrane integrity, body cell mass, and fluid distribution [12,13,14,15]. Recent systematic reviews and meta-analyses in critically ill populations consistently report lower PhA values in non-survivors, supporting its prognostic potential [15,16]. More recent cohort studies also suggest that longitudinal declines in PhA are associated with mortality in critically ill patients [17,18]. However, clinically applicable ICU-specific thresholds and the added predictive value of PhA beyond established severity scores remain uncertain.

Muscle ultrasound is another appealing bedside tool because it does not require patient transport, can be repeated over time, and allows direct assessment of peripheral skeletal muscle [19,20]. However, recent reviews have highlighted substantial methodological heterogeneity across ICU ultrasound studies, including differences in anatomical landmarks, probe pressure, patient position, and the muscle parameter selected for analysis [20,21,22,23]. These sources of variation limit the interpretation and generalizability of absolute cut-offs and complicate comparisons of prognostic performance across studies [22,24].

In this context, we hypothesized that a multimodal admission assessment integrating GLIM criteria, PhA, and rectus femoris ultrasound could improve early prognostic stratification in critically ill adults. We therefore evaluated the association of these nutritional and body composition markers with 30-day mortality. Continuous PhA and rectus femoris CSA were prioritized in multivariable analyses to avoid information loss and optimism related to cohort-derived dichotomization, whereas ROC-derived cut-offs were considered exploratory. Secondary aims were to describe malnutrition prevalence and explore associations with ICU length of stay.

## 2. Materials and Methods

### 2.1. Study Design and Setting

This prospective observational cohort study was conducted in the 12-bed adult intensive care unit of Punta de Europa University Hospital (Algeciras, Spain). Consecutive eligible patients admitted between September 2024 and March 2026 were prospectively enrolled in the study database. The present analysis evaluated admission nutritional assessment and 30-day outcomes.

### 2.2. Participants

**Figure 1:**
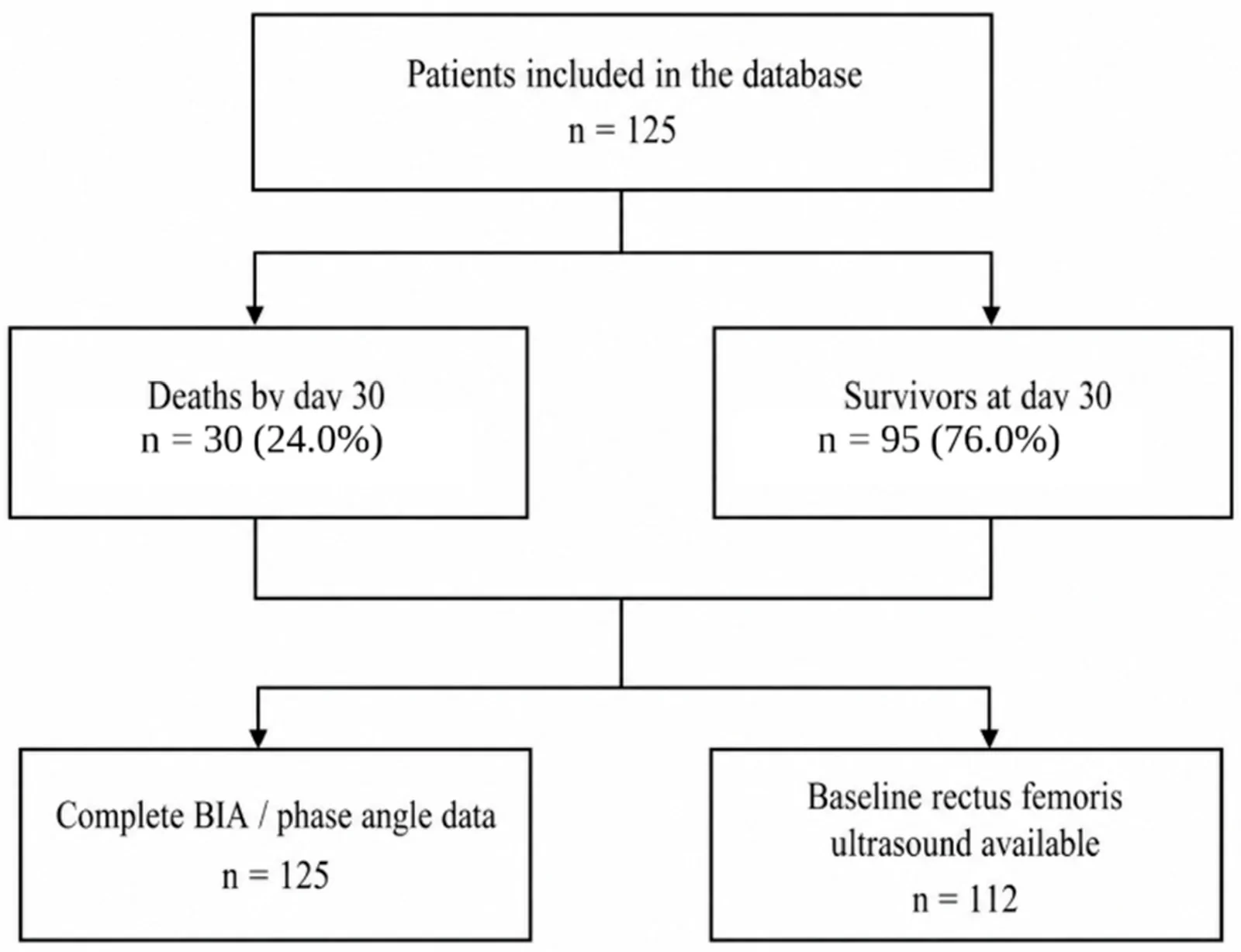
Study flow and 30-day vital status. Note: Total ICU admissions, numbers screened, pre-enrollment exclusions, refusals, and detailed exclusion reasons were not prospectively retained in a screening log and therefore cannot be reconstructed reliably.

Adults aged ≥ 18 years with an ICU stay >24 h were eligible. Baseline nutritional assessment was performed within the first 24–48 h after ICU admission. Patients with conditions precluding reliable BIA, particularly generalized edema/anasarca or other body alterations preventing electrode placement or valid whole-body measurement, were excluded. Fever, diuretic exposure, and isolated fluid/electrolyte disturbances were not among the applied exclusion criteria and were therefore not added retrospectively. Eligible patients who provided consent formed the analytic cohort (*n* = 125). A pre-enrollment screening log was not maintained; consequently, the total number of ICU admissions, patients screened, patients excluded, refusal counts, and detailed reasons for non-enrollment cannot be reconstructed reliably (Figure 1).

### 2.3. Nutritional and Body Composition Assessment

Baseline nutritional assessment included the Global Leadership Initiative on Malnutrition (GLIM) criteria [6], the Controlling Nutritional Status (CONUT) score [25], and the Spanish version of the SARC-F questionnaire [26]. GLIM diagnosis required at least one phenotypic criterion together with at least one etiologic criterion. Phenotypic thresholds were those specified in the study protocol: moderate weight loss criterion, 5–10% within the previous 6 months or 10–20% over more than 6 months, and severe weight loss criterion, >10% within 6 months or >20% over more than 6 months. Low-BMI thresholds for moderate malnutrition were <20 kg/m^2^ for patients < 70 years and <22 kg/m^2^ for patients ≥ 70 years; severe thresholds were <18.5 and <20 kg/m^2^, respectively. The etiologic reduced-intake/assimilation criterion comprised intake ≤50% of energy requirements, any reduction lasting >2 weeks, or a chronic gastrointestinal condition affecting assimilation/absorption; inflammation was defined by acute disease/injury or chronic disease-related inflammation. Overall GLIM severity was determined by the most severe phenotypic criterion present. The study protocol used a pragmatic two-step morphofunctional algorithm to operationalize the muscle-related phenotypic component. Handgrip strength was interpreted according to the age- and sex-stratified Spanish reference percentiles reported by Sánchez Torralvo et al. [27]. In the original study database, values < P10 were classified as marked muscle impairment without requiring additional BIA confirmation, whereas values between P10 and P50 (≥P10 to ≤P50) required low BIA-derived FFMI/SMI according to the age- and sex-referenced AKERN classification; values > P50 did not fulfill the protocol-specific muscle criterion. We acknowledge that handgrip strength is a measure of muscle function rather than muscle mass and, therefore, the <P10 rule should not be interpreted as direct confirmation of the GLIM reduced-muscle-mass phenotype. BIA-derived FFMI/SMI represented the quantitative muscle mass assessment available in this cohort [28]. Accordingly, the GLIM categories used in the primary analyses reflect the protocol-specific operationalization applied prospectively in the study. Rectus femoris ultrasound was acquired separately following the García-Almeida et al. protocol [24] and was not used to assign the GLIM muscle criterion. Accordingly, potential measurement-domain overlap exists between the GLIM muscle criterion and PhA because both can involve BIA-derived information, whereas there is no direct classification circularity between GLIM and rectus femoris CSA. This potential BIA/PhA overlap was addressed in sensitivity analyses excluding severe GLIM-defined malnutrition. Because interleukin-6 was not routinely available, the modified Nutrition Risk in the Critically Ill (mNUTRIC) score, which excludes interleukin-6, was used [8].

BIA was performed at the bedside using a 50 kHz whole-body analyzer (Akern BIA 101; Nutrilab^® ^ Akern S.r.l., Pontassieve, Tuscany, Italy) by the same trained investigator. Measurements were obtained with the patient supine using the standard wrist–ankle configuration with one electrode pair (bipolar arrangement), consistent with the bedside BIA methodology described by Mulasi et al. [28]. The right side was used whenever the right upper and lower limbs were anatomically suitable and were not being used for active intravenous infusion; an anatomically altered or actively infused limb was avoided for electrode placement. PhA was prespecified as the main BIA-derived variable of interest. Additional variables included total body water, extracellular water, fat-free mass, and FFMI/SMI-related measures. Derived body composition indices were obtained from the manufacturer-provided AKERN analysis system and interpreted according to the age- and sex-referenced categories used during the study. This is consistent with the broader BIA literature [28], which emphasizes population-, age-, and sex-related considerations in prediction equations and reference interpretation. Pre-measurement rest duration, number of replicate acquisitions, ventilation-specific adaptations, cumulative fluid balance, edema grade at the exact measurement time, circulatory status, and exact timing relative to systemic fluid boluses or diuretic administration were not systematically recorded as dedicated measurement time variables. These limitations were considered when interpreting PhA in critically ill patients.

Nutritional ultrasound was performed by the same trained investigator, following the standardized protocol of García-Almeida et al. [24]. Patients were examined supine, with the hip and knee extended and relaxed. The right side was used unless an anatomical limitation required contralateral measurement. The measurement point was located at the lower third of the line between the anterior superior iliac spine and the upper border of the patella. A linear broadband multifrequency transducer was used in the 5–10 MHz range recommended in the reference protocol, with frequency adjusted to optimize tissue penetration and image resolution. For CSA, the probe was kept perpendicular to the skin, and placement and contact pressure were standardized according to the published protocol to reduce technique-related variability. The rectus femoris area was obtained in transverse section by tracing the edge of the muscle aponeurosis. Three measurements were acquired, and their mean was recorded. No formal intra-rater reliability substudy or independent second-reader assessment was performed; the use of a single trained investigator minimized interobserver variation but does not provide a formal reliability estimate. Absolute CSA was analyzed as recorded; no post hoc normalization to height or BMI was introduced because it was not prespecified. Sex was retained in multivariable models, while residual confounding by body habitus is acknowledged. Baseline ultrasound data were available for 112 of the 125 participants.

### 2.4. Clinical Variables and Outcomes

Baseline clinical covariates included age, sex, body mass index (BMI), Acute Physiology and Chronic Health Evaluation II (APACHE II) score, Sequential Organ Failure Assessment (SOFA) score, need for invasive mechanical ventilation, and baseline albumin, prealbumin, and C-reactive protein levels. The primary endpoint was 30-day all-cause mortality. Secondary endpoints were ICU length of stay and total hospital length of stay, defined as days from hospital admission to discharge and including ICU stay.

### 2.5. Statistical Analysis

The distribution of continuous variables was assessed using visual inspection of histograms and the Shapiro–Wilk test. Continuous variables are presented as median and interquartile range [IQR] and categorical variables as n/N (%). Between-group comparisons were performed with the Mann–Whitney U test for continuous variables and the chi-square test or Fisher’s exact test for categorical variables, as appropriate. For 2 × 2 tables, the continuity-corrected chi-square test was used when the chi-square approach was applicable. Two-sided *p*-values < 0.05 were considered statistically significant. Because the primary analysis was prognostic and exploratory for some secondary endpoints, no formal multiplicity correction was applied to secondary comparisons.

ROC curves were generated for APACHE II, SOFA, mNUTRIC, CONUT, PhA, and rectus femoris CSA. AUCs were accompanied by 95% bootstrap confidence intervals, and optimal cut-offs were identified using the Youden index. These cohort-derived cut-offs were considered exploratory descriptors and were not used in the primary multivariable analysis. PhA and rectus femoris CSA were retained as continuous variables for adjusted analyses. Potential non-linearity was examined using restricted cubic splines with three knots placed at the 10th, 50th, and 90th percentiles and likelihood ratio comparison with the corresponding linear term; in the absence of evidence of non-linearity, linear terms were retained. For consistency with ROC operating characteristics, exploratory low-value thresholds used a ≤boundary (PhA ≤ 4.8° and CSA ≤ 3.51 cm^2^). ORs and aORs with 95% confidence intervals were calculated.

APACHE II, SOFA, and mNUTRIC were strongly collinear in this cohort (Spearman rho 0.76–0.85 among severity scores), so APACHE II was retained as the global severity adjustment variable. To respect the 30 outcome events and limit overfitting, the clinical reference logistic model included sex, APACHE II, and severe GLIM-defined malnutrition. The primary expanded model added continuous PhA, expressed per 1° lower value; a secondary ultrasound model added continuous rectus femoris CSA, expressed per 1 cm^2^ lower value, to the same covariate set. Incremental prognostic information from PhA was assessed using changes in AUC and the Brier score and a likelihood ratio test. To address potential overlap between the GLIM reduced-muscle-mass component and BIA/ultrasound exposures, sensitivity models were also repeated without severe GLIM-defined malnutrition. Cohort-derived binary cut-offs were examined only as exploratory sensitivity analyses; cut-off selection was not treated as part of confirmatory bootstrap validation. Model performance was summarized using AUC, AIC, the Brier score, and the Hosmer–Lemeshow test. Internal validation of the continuous PhA model used 1000 bootstrap resamples to estimate optimism-corrected discrimination. Complete-case analysis was used, and no multiple imputation was performed. Reporting followed TRIPOD recommendations and STROBE principles. Statistical analyses were performed using R software (2.12.2). To directly examine a reviewer-identified concern regarding the protocol-specific handgrip component, an additional post hoc sensitivity analysis was performed. For this analysis only, the binary severe GLIM predictor was coded positive only for participants originally classified as having severe GLIM-defined malnutrition who also had BIA-confirmed low SMI; participants with an original severe classification but without low BIA-derived SMI were coded as non-severe for this sensitivity definition. The same adjustment set (sex, APACHE II, and continuous PhA) was retained.

### 2.6. Ethics

The study was conducted in accordance with the Declaration of Helsinki and applicable Spanish regulations on biomedical research and data protection. The protocol was approved by the Provincial Research Ethics Committee of Cádiz (approval number: 230.23; approval date: 25 January 2024) . Written informed consent was obtained from all participants or, when necessary, from their legally authorized representatives prior to inclusion.

### 2.7. Declaration of Generative AI and AI-Assisted Technologies in the Writing Process

During preparation of this work, the authors used OpenAI ChatGPT (GPT-5.5 version) for grammar checks, spelling correction, refinement of academic style and support in figure preparation. The tool was not used for study design, data collection, statistical analysis or primary interpretation of the results. The authors reviewed and edited all AI-assisted content and take full responsibility for the content of the manuscript.

## 3. Results

The study cohort comprised 125 critically ill adults, of whom 79 (63.2%) were men. The median age was 68 [60–76] years, and the median BMI was 26.1 [23.9–29.7] kg/m^2^. Thirty-day all-cause mortality was 24.0% (30/125). GLIM-defined malnutrition affected 98 patients (78.4%), including 83 (66.4%) with moderate and 15 (12.0%) with severe malnutrition. Median baseline PhA was 4.6 [4.0–5.3] degrees, and baseline rectus femoris CSA was 3.3 [2.3–4.7] cm^2^ among the 112 participants with available ultrasound data. Figure 1 summarizes study flow and data availability.

Compared with survivors, non-survivors were older (75.0 [60.5–79.0] vs. 67.0 [60.5–74.0] years; *p* = 0.035) and had markedly greater illness severity, reflected by higher APACHE II, SOFA, and mNUTRIC scores (all *p* < 0.001), together with a higher need for invasive mechanical ventilation (66.7% vs. 26.3%; *p* < 0.001) (Table 1). Poorer nutritional status was also more frequent among non-survivors, including GLIM-defined malnutrition (93.3% vs. 73.7%; *p* = 0.043), severe GLIM-defined malnutrition (33.3% vs. 5.3%; *p* < 0.001), and lower albumin (*p* = 0.001). Non-survivors also had lower median PhA (4.3 [3.6–4.8] vs. 4.8 [4.2–5.5]°; *p* = 0.008) and smaller rectus femoris CSA (2.6 [1.9–3.4] vs. 3.6 [2.5–5.0] cm^2^; *p* = 0.007).

**Table 1:** Baseline characteristics according to 30-day vital status.

Variable	Overall (*n* = 125)	Survivors (*n* = 95)	Non-Survivors (*n* = 30)	*p*-Value
Age, years	68.0 [60.0–76.0]	67.0 [60.5–74.0]	75.0 [60.5–79.0]	0.035
Male sex	79 (63.2%)	59 (62.1%)	20 (66.7%)	0.815
BMI, kg/m^2^	26.1 [23.9–29.6]	26.0 [23.7–29.9]	26.5 [24.0–29.4]	0.924
APACHE II score	14.0 [9.0–19.0]	12.0 [8.0–16.0]	21.0 [16.3–26.8]	<0.001
SOFA score	3.0 [2.0–6.0]	2.0 [1.0–5.0]	8.0 [5.0–10.0]	<0.001
mNUTRIC score	3.0 [2.0–5.0]	3.0 [2.0–4.0]	5.0 [4.0–7.0]	<0.001
Mechanical ventilation	45 (36.0%)	25 (26.3%)	20 (66.7%)	<0.001
GLIM-defined malnutrition	98 (78.4%)	70 (73.7%)	28 (93.3%)	0.043
GLIM severe malnutrition	15 (12.0%)	5 (5.3%)	10 (33.3%)	<0.001
Moderate/severe CONUT	59 (47.2%)	37 (38.9%)	22 (73.3%)	0.002
High SARC-F probability	87 (69.6%)	60 (63.2%)	27 (90.0%)	0.011
Albumin, g/dL	3.0 [2.5–3.8]	3.5 [2.7–4.0]	2.7 [2.4–2.9]	0.001
C-reactive protein, mg/L	107.5 [30.7–220.8]	83.4 [24.1–184.5]	165.7 [75.9–261.4]	0.007
PhA, °	4.6 [4.0–5.3]	4.8 [4.2–5.5]	4.3 [3.6–4.8]	0.008
Low PhA (≤4.8°)	74 (59.2%)	49 (51.6%)	25 (83.3%)	0.004
Rectus femoris area, cm^2^	3.3 [2.3–4.7]	3.6 [2.5–5.0]	2.6 [1.9–3.4]	0.007
Low muscle area (≤3.51 cm^2^)	61/112 (54,5%)	40/86 (46.5%)	21/26 (80.8%)	0.004
Total body water, %	53.0 [48.9–57.0]	52.0 [48.6–56.4]	55.4 [52.1–60.0]	0.027

Data are median [IQR] or n/N (%). *p*-values compare survivors vs. non-survivors using the Mann–Whitney U test, continuity-corrected chi-square test, or Fisher’s exact test as appropriate. Denominators vary because of item-level missing data for some biochemical and ultrasound variables. Rows based on ROC-derived PhA and CSA thresholds are exploratory and use the same ≤boundary as the ROC operating characteristics in Table 2.

**Table 2:** Exploratory diagnostic performance of baseline markers for 30-day mortality.

Marker	*n*	AUC (95% CI)	Cut-Off	Se	Sp	PPV	NPV
APACHE II	124	0.837 (0.752–0.908)	17.00	0.733	0.755	0.489	0.899
SOFA	124	0.844 (0.759–0.916)	7.00	0.633	0.883	0.633	0.883
mNUTRIC	125	0.833 (0.745–0.910)	5.00	0.667	0.842	0.571	0.889
CONUT	103	0.739 (0.638–0.832)	4.00	0.920	0.487	0.365	0.950
PhA	125	0.660 (0.546–0.774)	4.80	0.833	0.484	0.338	0.902
Rectus femoris cross sectional area, cm^2^	112	0.674 (0.547–0.792)	3.51	0.808	0.535	0.344	0.902

AUC = area under the ROC curve; CI = confidence interval; NPV = negative predictive value; PPV = positive predictive value; Se = sensitivity; Sp = specificity. Cut-offs were identified with the Youden index in the same cohort and are therefore considered exploratory; they were not used in the primary multivariable analysis.

**Figure 2:**
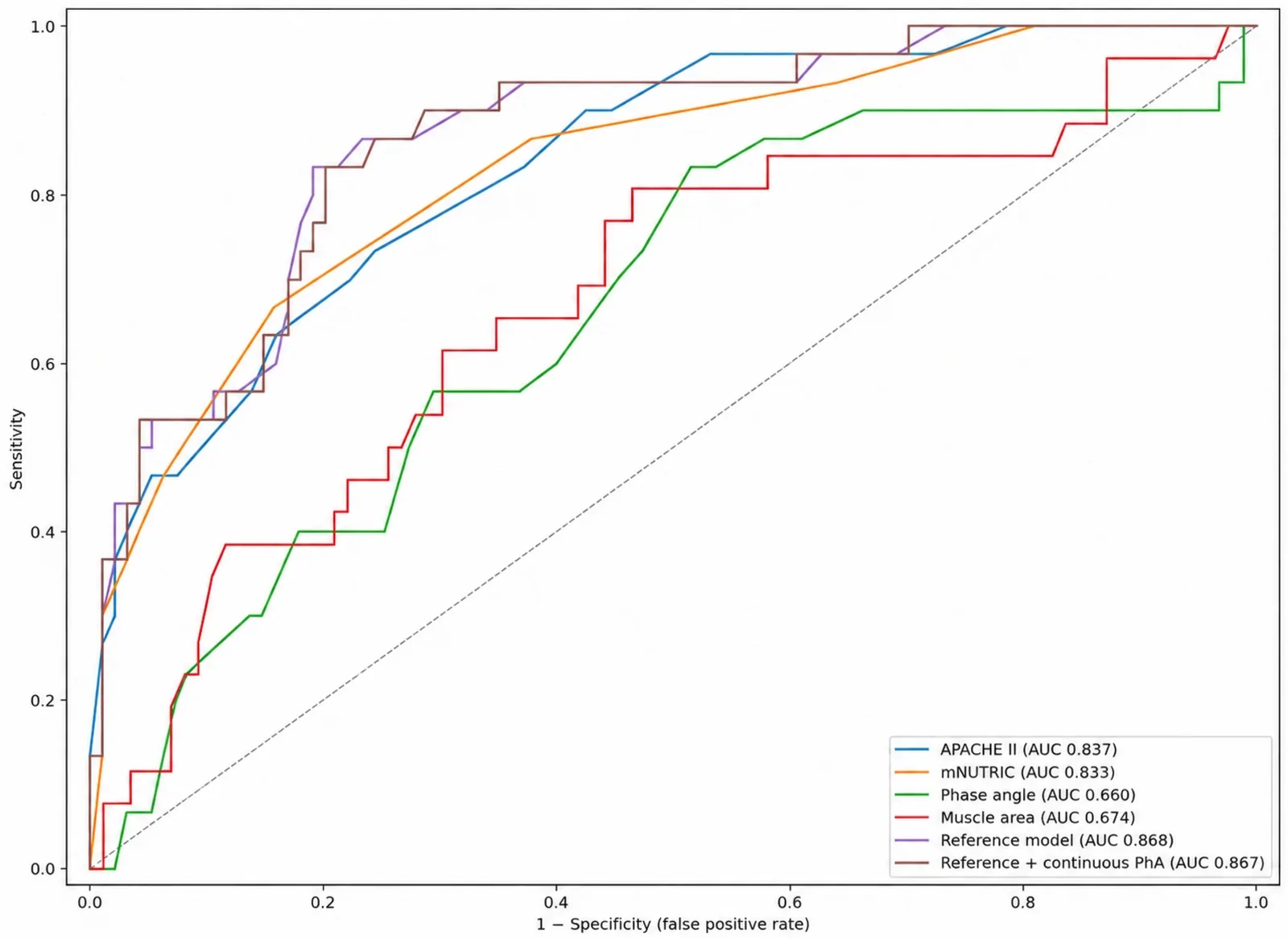
ROC curves for 30-day mortality. The reference model includes sex, APACHE II, and severe GLIM-defined malnutrition; the expanded model adds continuous admission PhA. The x-axis is 1 − specificity (false positive rate).

**Figure 3:**
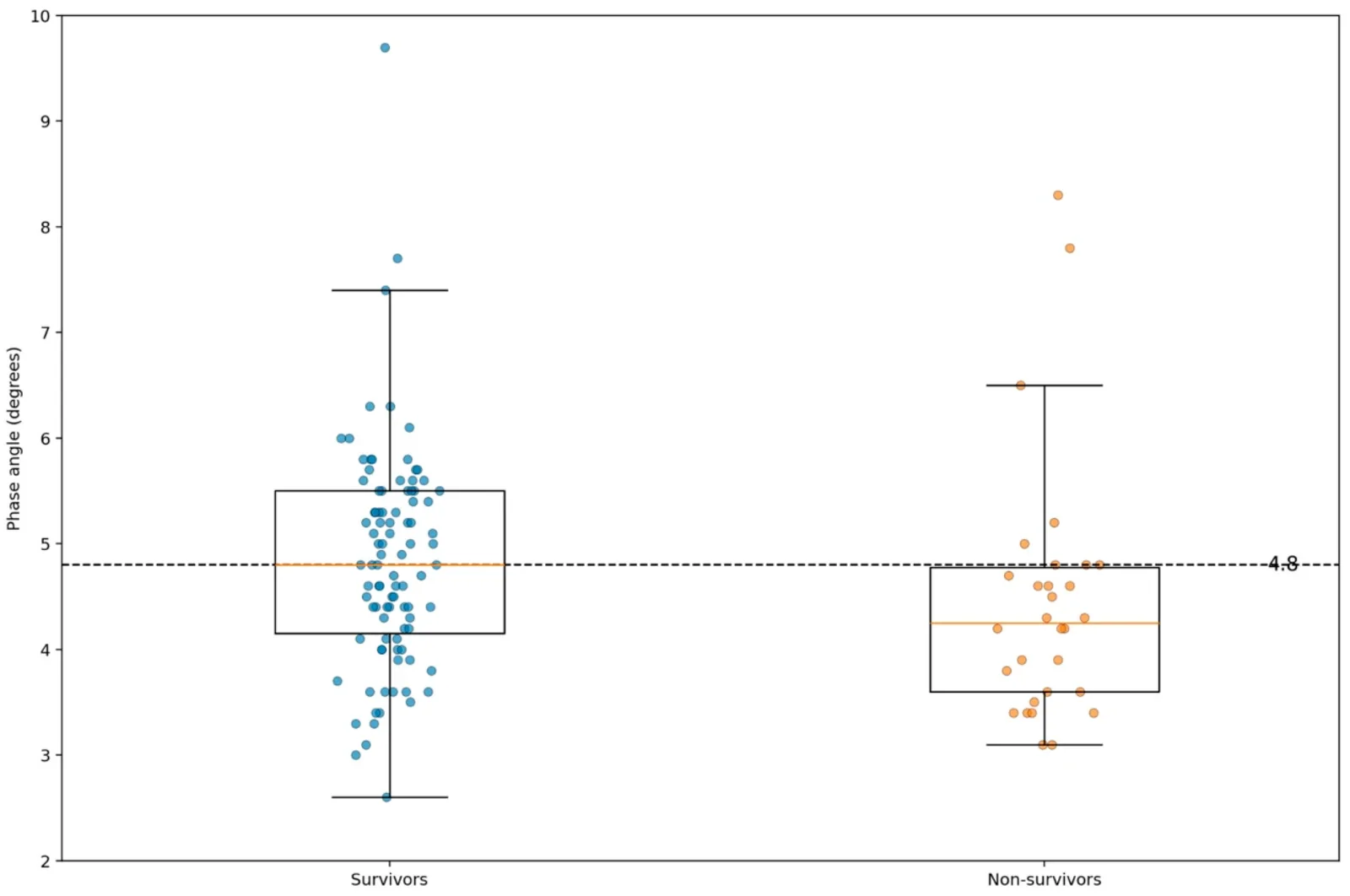
Distribution of baseline phase angle according to 30-day survival. The dashed line indicates the exploratory ROC-derived threshold of 4.8°.

**Figure 4:**
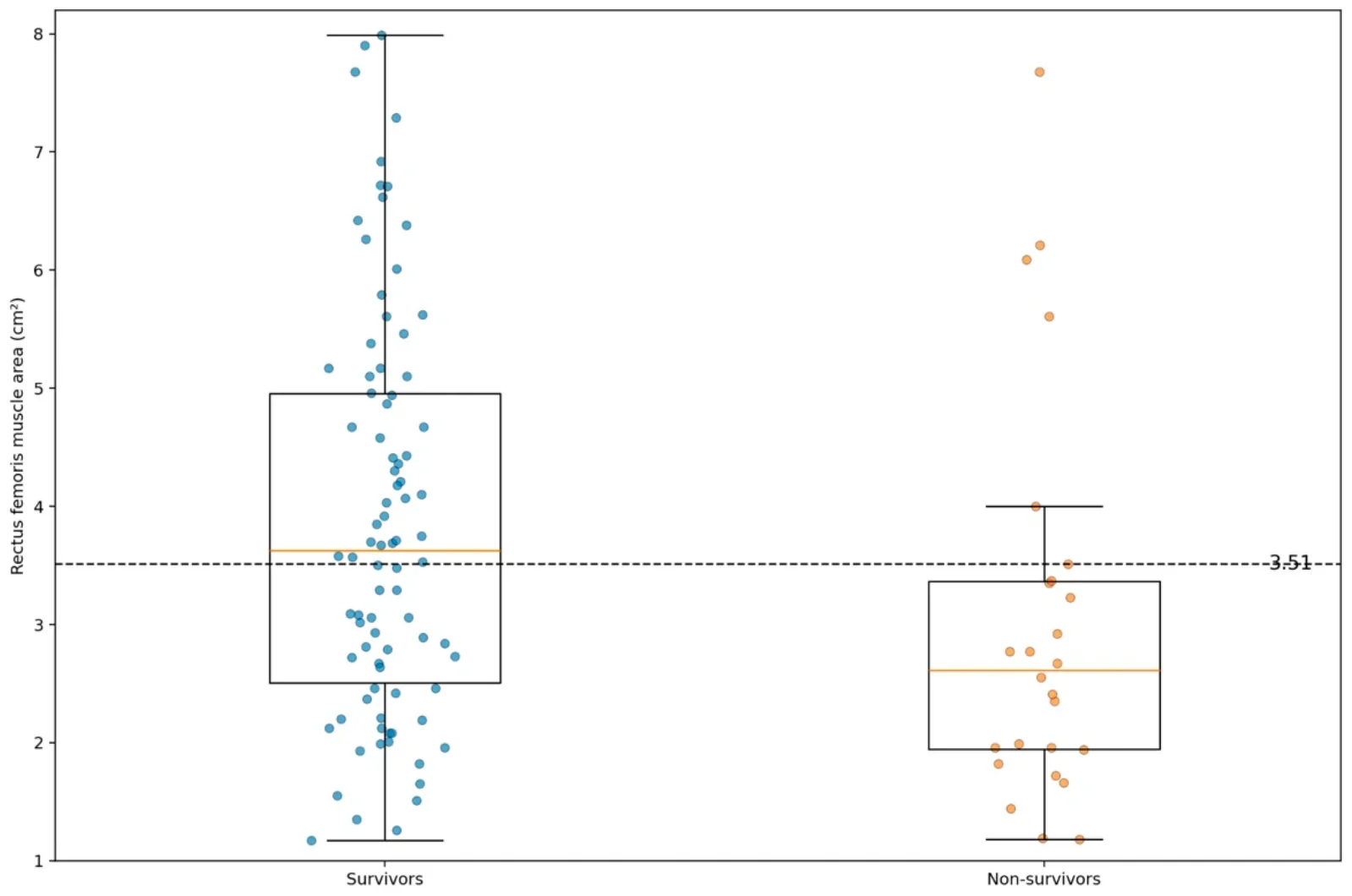
Distribution of baseline rectus femoris muscle area according to 30-day survival. The dashed line indicates the exploratory ROC-derived threshold of 3.51 cm^2^.

Among individual baseline markers, APACHE II, SOFA, and mNUTRIC showed the highest discrimination for 30-day mortality (AUCs 0.837, 0.844, and 0.833, respectively; Table 2; Figure 2). PhA and rectus femoris CSA showed more modest discrimination (AUC 0.660 and 0.674), with exploratory ROC-derived thresholds of 4.8° and 3.51 cm^2^, respectively. Because these thresholds were derived and evaluated in the same cohort, they were not used in the primary multivariable analysis. Figure 3 and Figure 4 show the distributions of PhA and rectus femoris CSA according to 30-day survival.

In the primary multivariable continuous PhA model (Table 3), APACHE II remained independently associated with 30-day mortality (OR 1.23 per point, 95% CI 1.12–1.35; *p* < 0.001), as did severe GLIM-defined malnutrition (OR 5.95, 95% CI 1.45–24.32; *p* = 0.013). In contrast, lower PhA was not independently associated with mortality when modeled continuously (OR 1.11 per 1° lower, 95% CI 0.74–1.69; *p* = 0.611). The continuous PhA model had AUC 0.867, Brier score 0.118, AIC 102.2, and Hosmer–Lemeshow *p* = 0.704; its optimism-corrected AUC was 0.843 after 1000 bootstrap resamples. The reference model containing sex, APACHE II, and severe GLIM-defined malnutrition had AUC 0.868, Brier score 0.118, and AIC 100.4. Adding continuous PhA changed the AUC by approximately −0.001 and the Brier score by −0.0002, with no evidence of improved model fit (likelihood ratio *p* = 0.607). There was no evidence of non-linearity for PhA (*p* = 0.636).

In the exploratory continuous ultrasound model (Table 3), APACHE II remained independently associated with mortality (OR 1.22 per point, 95% CI 1.11–1.33; *p* < 0.001). Severe GLIM-defined malnutrition showed a large but imprecise association that narrowly missed conventional statistical significance (OR 4.26, 95% CI 0.97–18.73; *p* = 0.055), while lower rectus femoris CSA was not independently associated with mortality (OR 1.17 per 1 cm^2^ lower, 95% CI 0.81–1.69; *p* = 0.412). Model AUC was 0.857, the Brier score was 0.119, AIC was 93.7, and Hosmer–Lemeshow *p* = 0.730. There was no statistically significant evidence of non-linearity for CSA (*p* = 0.108).

Exploratory sensitivity analyses based on cohort-derived thresholds remained boundary- and model-dependent. Using the same ≤convention as the ROC operating characteristics, PhA ≤ 4.8° had an adjusted OR of 4.16 (95% CI 1.11–15.59; *p* = 0.035), whereas rectus femoris CSA ≤ 3.51 cm^2^ had an adjusted OR of 3.43 (95% CI 0.98–11.98; *p* = 0.053). Because the cut-offs were selected in the same cohort, these estimates were considered exploratory and were not used to support independent prognostic claims. To address potential measurement-domain overlap between the BIA-supported GLIM reduced-muscle-mass criterion and BIA-derived PhA, continuous models were repeated without severe GLIM-defined malnutrition; lower PhA remained non-significant (OR 1.19 per 1° lower, 95% CI 0.78–1.82; *p* = 0.427), as did lower CSA (OR 1.23 per 1 cm^2^ lower, 95% CI 0.86–1.77; *p* = 0.250). In secondary descriptive analyses, median ICU and hospital length of stay were 7.0 [4.0–10.0] and 13.0 [7.0–19.0] days. Patients with PhA ≤ 4.8° had longer ICU stays than those with PhA > 4.8° (7 [5–11] vs. 5.5 [3–9.8] days; *p* = 0.029), while hospital length of stay did not differ significantly (*p* = 0.252). These post-baseline outcomes were interpreted descriptively because death is a competing event.

The protocol-specific handgrip and BIA classifications were not interchangeable. Among 86 participants with handgrip strength <P10, 37 (43.0%) also had low BIA-derived SMI, whereas 49 (57.0%) did not. In a post hoc sensitivity analysis restricting the severe GLIM predictor to the eight participants whose original severe GLIM classification coincided with low BIA-derived SMI, the association with 30-day mortality remained statistically significant (adjusted OR 14.15, 95% CI 1.81–110.95; *p* = 0.012), while continuous PhA remained non-significant (adjusted OR 1.07 per 1° lower, 95% CI 0.69–1.64; *p* = 0.768). This analysis included 124 complete cases and 30 deaths. Because only eight participants met this stricter BIA-confirmed severe GLIM sensitivity definition, the confidence interval was wide, and the analysis was considered exploratory.

## 4. Discussion

This prospective cohort study yields three main findings. First, malnutrition at ICU admission was highly prevalent, with nearly four in five patients meeting GLIM criteria and 12% presenting severe malnutrition. Second, non-survivors had lower admission PhA, but continuous PhA did not retain an independent association with 30-day mortality after adjustment for sex, APACHE II, and severe GLIM-defined malnutrition and did not improve the clinical reference model. Third, rectus femoris CSA was lower among non-survivors in unadjusted analyses but was not independently associated with mortality when modeled continuously. Severe GLIM-defined malnutrition remained the nutritional variable most consistently associated with 30-day mortality in the primary adjusted model.

The high prevalence of GLIM-defined malnutrition in our cohort aligns with growing ICU evidence that nutritional impairment is common during critical illness. Milanez et al. (2023) demonstrated that GLIM assessment is feasible and frequently positive in critically ill adults [9]. Foletto et al. (2024) reported improved prognostic stratification when GLIM criteria were combined with nutrition risk screening tools [10]. Kim et al. (2025) found that an integrated mNUTRIC–GLIM approach outperformed several isolated markers for in-hospital mortality [11], while Liu et al. (2024) associated GLIM-defined malnutrition with adverse outcomes in neurocritical care [29]. In this context, our results suggest that severe GLIM-defined malnutrition, rather than GLIM positivity alone, is the component most strongly associated with 30-day mortality.

The GLIM finding requires specific caution regarding the muscle phenotype. The original study protocol incorporated handgrip strength into a pragmatic morphofunctional algorithm, and values <P10 could satisfy the protocol-specific muscle criterion without BIA confirmation. Handgrip strength reflects muscle function rather than muscle mass, and the present data support keeping these constructs distinct: only 37 of 86 participants (43.0%) with handgrip <P10 also had low BIA-derived SMI. We therefore do not interpret very low handgrip strength alone as confirmation of reduced muscle mass. Importantly, when the severe GLIM predictor was restricted in a post hoc sensitivity analysis to the eight originally severe cases with BIA-confirmed low SMI, its association with 30-day mortality persisted, whereas continuous PhA remained non-significant. The very wide confidence interval in this sensitivity analysis reflects the small number of BIA-confirmed severe cases; therefore, this result supports qualitative robustness of the association but does not validate the original operational definition, which requires confirmation in cohorts using strictly mass-based GLIM muscle criteria.

Our unadjusted findings regarding PhA are consistent with evidence from critical care populations linking lower PhA with mortality [16]. Non-survivors in the present cohort had lower PhA than survivors. However, the revised continuous analysis did not demonstrate an independent association after adjustment, and restricted cubic spline analysis did not identify evidence of non-linearity. Accordingly, the cohort-derived 4.8° threshold should be interpreted as exploratory rather than as a validated prognostic boundary, even though the dichotomized sensitivity model produced a statistically significant estimate after correction of the 30-day endpoint.

When continuous PhA was added to a reference model containing sex, APACHE II, and severe GLIM-defined malnutrition, the AUC changed from 0.868 to 0.867, the Brier score improved only minimally, and the likelihood ratio test was not significant. This distinction is important because dichotomizing a continuous biomarker at a cut-off selected from the same cohort can amplify apparent effects and produce unstable estimates, particularly with a limited number of events. The exploratory binary PhA association should therefore not override the null continuous and incremental performance analyses. Longitudinal changes in PhA may still be informative, as reported in other critical care cohorts [18,30], but this question could not be robustly addressed with the incomplete follow-up measurements in the present study.

The findings related to rectus femoris ultrasound should also be interpreted cautiously. Smaller admission CSA was associated with mortality in unadjusted analyses, which is biologically plausible and consistent with evidence linking reduced skeletal muscle reserve at ICU admission with adverse outcomes [19,31]. However, neither continuous CSA nor the exploratory dichotomized threshold provided robust evidence of an independent association after adjustment. A sensitivity model excluding severe GLIM-defined malnutrition yielded the same qualitative conclusion. Importantly, rectus femoris CSA was not used in the operational rule that defined the GLIM reduced-muscle-mass criterion in this cohort, so there was no direct classification circularity between the ultrasound exposure and GLIM muscle status. Thus, in this cohort, rectus femoris ultrasound appears more useful as a bedside phenotyping measure than as an independently validated mortality predictor.

Methodological factors may partly explain these findings. Ultrasound measurements vary according to anatomical landmark, patient position, body size, and probe pressure [21,22,23]. We followed a standardized García-Almeida protocol [24], using a fixed landmark, a supine relaxed position, right-sided measurements unless anatomically precluded, perpendicular transverse acquisition, three measurements, and the mean value. Nevertheless, no independent second-reader assessment or formal intra-rater reliability study was performed, so measurement reliability cannot be quantified in this cohort. Absolute CSA was analyzed as prespecified and was not normalized post hoc to height or BMI; although sex was retained in adjusted models, residual confounding by body habitus remains possible. Repeated longitudinal ultrasound may better characterize rapid ICU muscle loss than a single baseline assessment [32,33,34].

From a clinical perspective, the findings support a layered approach to early ICU phenotyping while also defining the limits of the present data. APACHE II and SOFA quantify global illness severity, whereas GLIM and mNUTRIC provide complementary nutritional information. PhA offers a rapid bedside signal related to cellular integrity and fluid distribution [12], and rectus femoris ultrasound provides direct structural information about peripheral muscle. However, the present continuous analyses do not establish that either PhA or CSA adds independent mortality prediction beyond the clinical reference model. Their most defensible role in this cohort is therefore complementary phenotyping and hypothesis generation rather than stand-alone prognostic classification.

This study has several important strengths, including its prospective design, standardized baseline data collection and multimodal bedside nutritional assessment integrating clinical scores, GLIM criteria, bioelectrical impedance, handgrip assessment and muscle ultrasound. The revision also evaluates continuous associations, incremental model performance, calibration, sensitivity analyses addressing measurement overlap, and internal bootstrap validation.

Several limitations should be acknowledged. This was a single-center study with 30 deaths, limiting precision and increasing the risk of unstable estimates when multiple predictors or data-derived cut-offs are examined. A pre-enrollment screening log was not maintained, so the total number of ICU admissions screened, numbers excluded or refusing participation, and reasons for non-enrollment cannot be quantified; selection bias related to early death, clinical instability, or inability to undergo bedside measurements therefore cannot be excluded. Admission PhA is sensitive to fluid distribution, and bioimpedance measurements in critically ill patients may be substantially affected by acute changes in hydration status [12,28,35]. Although BIA-derived hydration variables were available, cumulative fluid balance, edema grade, circulatory status at the exact measurement time, and exact timing relative to systemic fluid or diuretic administration were not systematically recorded as measurement time covariates. Right-sided wrist–ankle electrode placement and avoidance of limbs with anatomical alterations or active intravenous infusion were standardized, but pre-measurement rest duration, repeat-acquisition count, and ventilation-specific adaptations were not captured as dedicated database fields. These issues limit separation of nutritional and fluid-related determinants of PhA.

Ultrasound data were unavailable in 13 patients, including nine survivors and four non-survivors. Although missingness was not confined to non-survivors, the small number of patients without ultrasound precludes excluding selection bias. All ultrasound measurements were made by the same trained investigator according to a standardized protocol, but no intra-rater reliability statistic or independent second-reader assessment was obtained. Absolute CSA was not normalized to height or BMI, and residual body size effects remain possible. An important limitation is the protocol-specific operationalization of the GLIM muscle component. In particular, handgrip <P10 was originally accepted as marked muscle impairment without mandatory BIA confirmation, although handgrip measures function rather than muscle mass. The incomplete concordance with BIA-derived low SMI in this cohort confirms that these measures should not be treated as equivalent. We therefore distinguish the original protocol-defined GLIM classification from BIA-confirmed low muscle mass and interpret the severe GLIM association cautiously. The additional BIA-confirmed sensitivity analysis was directionally consistent but imprecise because only eight participants met the stricter definition. Because BIA-derived indices could contribute to the protocol-specific GLIM muscle criterion and PhA was obtained from the same BIA acquisition, some measurement-domain overlap with PhA remains possible. Rectus femoris CSA was not used to classify the GLIM muscle criterion, so direct circularity with the ultrasound exposure was avoided. We also lacked a gold-standard body composition method such as CT or DXA, and follow-up PhA/ultrasound data were too incomplete for robust longitudinal modeling. Finally, PhA and CSA thresholds were selected in the same cohort and should be considered hypothesis-generating until externally validated.

## 5. Conclusions

In critically ill adults, APACHE II and protocol-defined severe GLIM malnutrition retained independent associations with 30-day mortality in the primary adjusted model. Because the original muscle criterion incorporated handgrip strength, this GLIM association should be interpreted cautiously; a post hoc sensitivity analysis restricting the severe GLIM exposure to participants with BIA-confirmed low SMI yielded the same qualitative association but with substantial imprecision. Admission PhA and rectus femoris CSA were lower among non-survivors but did not independently contribute when modeled continuously after adjustment. The cohort-derived ROC thresholds for PhA and CSA remain exploratory despite the association observed for dichotomized PhA in sensitivity analysis. Larger multicenter studies should validate continuous and externally defined thresholds, incorporate standardized fluid-status assessment and reproducible BIA/ultrasound protocols, and determine whether longitudinal changes in PhA and muscle ultrasound improve risk stratification and nutrition-focused phenotyping in critical care.

## Figures and Tables

**Table 3:** Multivariable logistic regression models for 30-day mortality.

Predictor	Primary Continuous PhA Model OR (95% CI)	*p*-Value	Continuous Ultrasound Model OR (95% CI)	*p*-Value
Male sex	1.76 (0.57–5.44)	0.328	1.82 (0.56–5.90)	0.320
APACHE II (per point)	1.23 (1.13–1.34)	<0.001	1.22 (1.11–1.33)	<0.001
GLIM severe malnutrition	5.95 (1.45–24.32)	0.013	4.26 (0.97–18.73)	0.055
PhA (per 1° lower)	1.11 (0.74–1.69)	0.611	—	—
Rectus femoris CSA (per 1 cm^2^ lower)	—	—	1.17 (0.81–1.69)	0.412

Primary continuous PhA model: *n* = 124 because one patient had missing APACHE II data; all 30 deaths were retained. Continuous ultrasound model: *n* = 111 because ultrasound was available in 112 participants and one additional patient had missing APACHE II data; 26 deaths were included. ORs for PhA and rectus femoris CSA are expressed per 1-unit decrease. Primary PhA model: AUC = 0.867, optimism-corrected AUC = 0.843 (1000 bootstrap resamples), Brier score = 0.118, AIC = 102.2, Hosmer–Lemeshow *p* = 0.704. Reference model: AUC = 0.868, Brier score = 0.118, AIC = 100.4; adding continuous PhA did not improve fit (likelihood ratio *p* = 0.607). Continuous ultrasound model: AUC = 0.857, Brier score = 0.119, AIC = 93.7, Hosmer–Lemeshow *p* = 0.730. Em dashes indicate predictors not included in the corresponding model.

## Data Availability

De-identified data may be made available by the corresponding author on reasonable request, subject to institutional and ethics requirements.

## Abbreviations

APACHE II	Acute Physiology and Chronic Health Evaluation II
AIC	Akaike information criterion
aOR	Adjusted odds ratio
AUC	Area under the receiver operating characteristic curve
BIA	Bioelectrical impedance analysis
BIVA	Bioelectrical impedance vector analysis
BMI	Body mass index
CI	Confidence interval
CONUT	Controlling Nutritional Status
CSA	Cross-sectional area
GLIM	Global Leadership Initiative on Malnutrition
ICU	Intensive care unit
IQR	Interquartile range
mNUTRIC	Modified Nutrition Risk in the Critically Ill
NPV	Negative predictive value
OR	Odds ratio
PhA	Phase angle
PPV	Positive predictive value
ROC	Receiver operating characteristic
SARC-F	Strength, Assistance with walking, Rise from a chair, Climb stairs, and Falls
Se	Sensitivity
SOFA	Sequential Organ Failure Assessment
Sp	Specificity
TRIPOD	Transparent Reporting of a multivariable prediction model for Individual Prognosis Or Diagnosis
